# Age, sex and race distribution of accelerometer-derived sleep variability in US school-aged children and adults

**DOI:** 10.21203/rs.3.rs-2927692/v1

**Published:** 2023-05-19

**Authors:** Elexis Price, Xinyue Li, Yanyan Xu, Asifhusen Mansuri, William V. McCall, Shaoyong Su, Xiaoling Wang

**Affiliations:** Augusta University; City University of Hong Kong; Georgia Prevention Institute, Augusta University; Children’s Hospital of Georgia, Augusta University; Augusta University; Georgia Prevention Institute, Augusta University; Georgia Prevention Institute, Augusta University

## Abstract

**Background:**

Sleep variability (e.g. intra-individual variabilities in sleep duration or sleep timing, social jetlag, and catch-up sleep) is an important factor impacting health and mortality. However, limited information is available on the distribution of these sleep parameters across the human life span. We aimed to provide distribution of sleep variability related parameters across lifespan by sex and race in a national representative sample from the U.S. population.

**Methods:**

The study included 9,799 participants 6 years and older from the National Health and Nutrition Examination Survey (NHANES) 2011–2014, who had at least 3 days of valid sleep parameters with at least one day obtained during weekend (Friday or Saturday night). These were calculated from 7-day 24-h accelerometer recordings.

**Results:**

Of the study participants, 43% showed ≥ 60 minutes sleep duration standard deviation (SD), 51% experienced ≥ 60 minutes catch-up sleep, 20% showed ≥ 60 minutes midpoint of sleep SD, and 43% experienced ≥ 60 minutes social jetlag. American youth and young adults averaged greater sleep variability compared to other age groups. Non-Hispanic Blacks showed greater sleep variability in all parameters compared to other racial groups. There was a main effect of sex on sleep midpoint SD and social jetlag with males averaging slightly more than females.

**Conclusion:**

Our study provides important observations on sleep irregularity parameters of residents of the United States by using objectively measured sleep patterns and will provide unique insights for personalized advice on sleep hygiene.

## INTRODUCTION

Adequate sleep is vital to overall human health and the importance of sufficient sleep duration is well-known^1^. However, recent research has begun to show that sleep variability, in terms of duration and timing, is also critical to optimal health^2^. Modern society commonly experiences considerable interference in sleep due to lifestyle demands which vary from day to day^3^. Furthermore, the constraints of school/work schedules and individual chronotypes drive differences in sleep patterns on weekdays compared to weekends when individuals typically try to “catch up on sleep”^3^. The difference of sleep timing on weekdays vs weekends is known as social jetlag. The variation of sleep duration and sleep timing from day to day can also be measured by the standard deviation of sleep duration (sleep duration SD) and the standard deviation of midpoint of sleep (midpoint of sleep SD). Inconsistency in sleep patterns has been linked to numerous negative health outcomes including cardiovascular disease, metabolic disruptions, immune system regulation, and many other adverse health conditions^4–7^. For example, large variability in sleep duration (> 1 h of sleep duration SD) has been associated with an increased risk of cardiovascular disease in older adults and suicidal ideation in young adults^8 4^. The protective effects of catch-up sleep on health have also been found to be offset in individuals with habitual increased sleep variability^9^.

The variability of sleep patterns changes with age, and a nationally representative sample is required to estimate these changes across the lifespan unbiasedly, including possible modifying demographic factors such as sex and race. A recent nationally representative cross-sectional analysis which assessed the prevalence of social jetlag among US adults using self-report questionnaire data found that almost half averaged at least 1h of social jetlag^10^, indicating the high prevalence of sleep irregularity in US adults. However, national survey data on objective measurements of sleep variability is limited. More recently, increasing the usage of rest activity data provided by research-grade activity monitors (i.e. accelerometers) in population studies allows for more objective estimates of sleep parameters that can be used to measure multiple parameters of sleep variability. In the current study, using nationally representative accelerometer data from the National Health and Nutrition Examination Survey (NHANES) from 2011–2014, we aimed to evaluate the distribution of sleep variability across the lifespan using objectively obtained multiple sleep variability parameters in 9,799 participants aged 6 years and older.

## METHOD

### Study population

**Figure 1:**
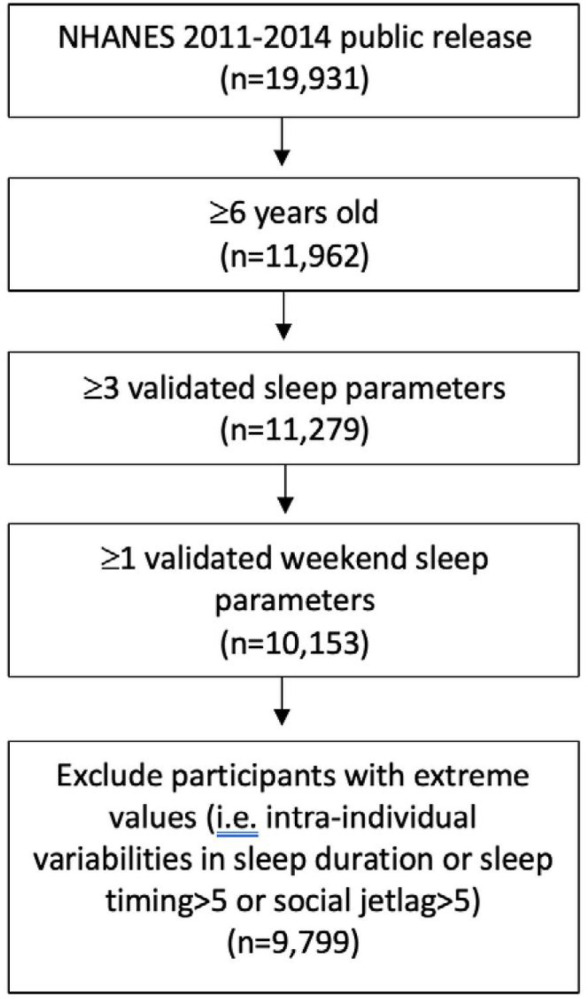
Flowchart for inclusion of study participants.

This study used data from the NHANES survey. The survey is the collection of health examination data for a weighted nationally representative sample of the U.S. population through a multistage probability sampling design^11^. The survey consists of questionnaires administered at the participants home followed by a health examination in a mobile examination center^11^. Participants receive compensation and a report of their medical findings^11^. The NHANES surveys from the 2011–2012 and 2013–2014 were used. These cycles were selected due to their availability of 24h accelerometer data. The National Center for Health Statistics Research Ethics Review Board approved all NHANES protocols, and written informed consent was obtained from all participants or their parents or guardians. All methods were performed in accordance with the relevant guidelines and regulations. Our sample included participants ≥ 6 years old from the NHANES 2011–2014, who had at least 3 days of valid sleep parameters with at least one day obtained during weekend (Friday or Saturday night) (*n* = 9,799). NHANES does not specify participant’s age after 80 years old for participant privacy, so all participants over 80 were coded as 80 years old. Figure 1 illustrates the flow chart for participants selected for inclusion within the analysis.

### Sleep parameters

Sleep parameters were derived from accelerometer data. Accelerometer recordings and data preprocessing were documented previously^12^. The calculation of sleep onset time, wakeup time, and sleep duration from the accelerometer data has also been described previously. Briefly, a Hidden Markov Model (HMM)-based unsupervised sleep-wake identification algorithm was used to infer the sequence of “hidden states” of sleep or wake for each individual^13^. The block of the longest sleep period in the day (noon-noon) was identified as the sleep period time (SPT) window. The start of the SPT window was defined as the sleep onset time and the end of the SPT window was defined as the wakeup time. Wake/activity bouts were identified during the SPT window. Sleep duration was defined using the following equation: sleep duration = the SPT window duration -the summed duration of all wake bouts. The midpoint of sleep was assessed by the sleep onset time and wakeup time. The variability parameters for sleep duration included the standard deviation of sleep duration (sleep duration SD) and catch-up sleep which was estimated as the absolute value of the difference in sleep duration between weekdays and weekends. The variability parameters for timing of sleep included the standard deviation of the midpoint of sleep (midpoint of sleep SD) and social jetlag which was estimated as the absolute value of the difference in midpoint of sleep times between weekdays and weekends. Standard deviation of sleep onset time (sleep onset SD) and standard deviation of wakeup time (wakeup SD) were also included as secondary variables for the sleep timing variability parameters.

### Age, sex, and race

The NHANES demographic file was used to obtain age, sex, and race information. Participants were divided into 9 different age groups for the description of the distributions of sleep variability parameters. Race was divided into 4 classifications: Non-Hispanic (NH) White, NH Black, Mexican American, and other race (i.e. other Hispanic, Asian and other ethnicity).

### Statistical analysis

Analyses were completed using the survey data analysis in STATA (v16) to generate representative estimates of the US population and account for complex survey design. Four-year survey weights were calculated and used in all analyses to adjust for unequal selection probability and non-response bias in accordance with NHANES analytical guidelines^14^. Population means, proportions, and standard deviation (SD) were estimated and reported. Survey weighted linear regression was used to assess the distribution of sleep variability parameters across age, sex and race. Due to the nonlinear relationship between age and sleep variability parameters, linear, quadratic, cubic and quartic trends were fitted by including age, age^2^, age^3^ and age^4^ in the regression model to understand the changes of sleep variability parameters with age. The interactions between sex and age (i.e. linear, quadratic, cubic and quartic) or between race and age were also tested to examine whether the associations of sleep variability parameters with age were modified by sex or race. Statistical significance was set at *p* < 0.05.

## RESULTS

The complete dataset consisted of 19, 931 participants and a total of 10,132 participants were excluded from analysis based on exclusion criteria (Fig. 1). Our final sample size included 9,799 participants aged ≥ 6 years (mean ± SD: 41.94 ± 21.51 years), representing 175.5 million noninstitutionalized residents of the United States. The general characteristics of age, sex, race, and sleep variability parameters of participants are presented in Table 1.

The distributions of sleep duration SD, catch-up sleep, midpoint of sleep SD and social jetlag by age groups are presented in Table 2 and Table 3. The medians ranged from 42 to 67 minutes for sleep duration SD, 49 to 78 minutes for catch-up sleep, 31 to 46 minutes for midpoint of sleep SD and 32 to 82 minutes for social jetlag across the age groups, with the peak values shown either in the age group of 14–17 or the age group of 18–25. In the overall sample, 43% of US school-aged children and adults showed ≥ 60 minutes sleep duration SD, 51% experienced ≥ 60 minutes catch-up sleep, 20% showed ≥ 60 minutes midpoint of sleep SD, and 43% experienced ≥ 60 minutes social jetlag. The prevalence of sleep irregularity defined by the cutoff of 60 minutes, 90 minutes and 120 minutes by these 4 sleep variability parameters by age groups are presented in supplementary Table 1 and Table 2.

**Figure 2:**
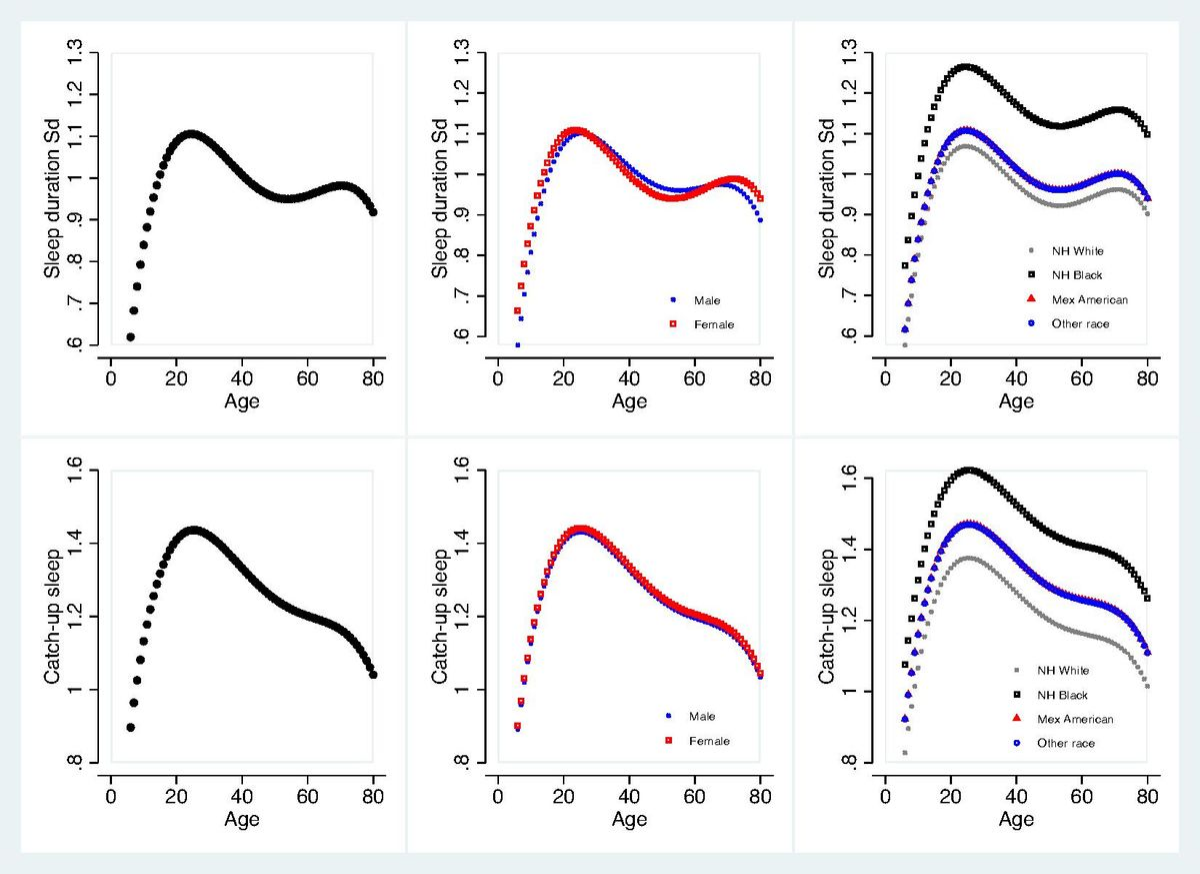
Age, sex, and race distribution of sleep duration SD and catch-up sleep. The unit for Y-axis is hours. (A) 1–3 for sleep duration SD. Please note the curve for Other race is overlapped with the curve of Mexican American. (B) 1–3 for catch-up sleep.

Figure 2 displays the changes in the sleep duration SD and catch-up sleep with age and possible effect modifiers of sex and race. Sleep duration SD increased until its first peak around age 20 then gradually declined until around age 50. After age 50, it showed a small increase again until around 70 to where it declined once more (Fig. 2A-1). There was no overall main effect of sex on sleep duration SD but there was a significant interaction between sex and age^2^. As shown in Fig. 2A-2, the decline in sleep duration SD from around age 20 to age 40 was larger in females. Furthermore, there was a significant main effect of race (*p* < 0.001) as NH blacks had the highest median sleep duration SD of 64 m compared to 53 m *(p* < 0.001), 54 m (*p* < 0.001), and 54 m (*p* < 0.001) for NH whites, Mexican Americans, and other races respectively. There were no significant interactions between race and age observed for sleep duration SD.

**Figure 3:**
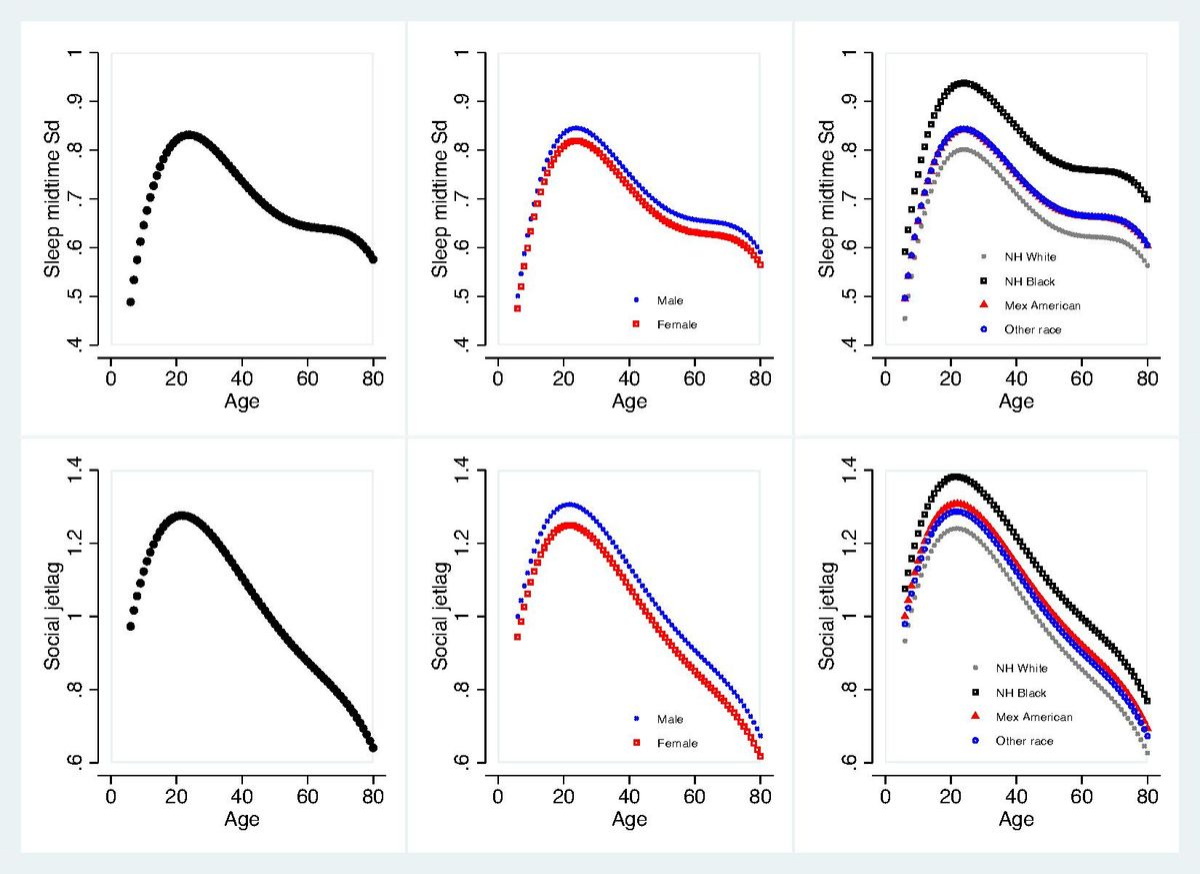
Age, sex and race distribution of midpoint of sleep SD and social jetlag. The unit for Y-axis is hours. (A) 1–3 for midpoint of sleep SD. Please note the curve for Other race is overlapped with the curve of Mexican American. (B) 1–3 for social jetlag.

Catch-up sleep followed a similar pattern as sleep duration SD as it increased till around age 20 then gradually declined throughout with a small plateau between ages 50 and 70 (Fig. 2B-1). There was no overall main effect of sex on catch-up sleep and there were no significant interactions between sex and age observed (Fig. 2B-2). However, there was a significant main effect of race (*p* < 0.001). NH blacks had the highest median catch-up sleep of 72 m compared to 57 m *(p* < 0.001), 65 m (*p* = 0.003), and 65 m (*p* = 0.022) for NH whites, Mexican Americans, and other races respectively (Fig. 2B-3). In addition, the difference between Mexican American and NH whites in catch-up sleep also reached significance (*p* = 0.027). There were no significant interactions between race and age observed for catch-up sleep.

Figure 3 displays the changes in the midpoint of sleep SD and social jetlag with age and possible effect modifiers of sex and race. As shown in Fig. 3A-1, midpoint of sleep SD increased with age until its peak around age 20. After age 20, midpoint of sleep SD gradually declined until around age 50 where it leveled off until around age 70 before declining once again. There was a significant main effect of sex (*p* = 0.013) with males averaging 2 more minutes of midpoint of sleep SD compared to females (Fig. 3A-2). There was also a significant main effect of race (*p* < 0.001) observed with NH blacks averaging 9 more minutes of midpoint of sleep SD compared to NH whites (*p* < 0.001) and 4 more minutes compared to Mexican Americans (*p* < 0.001) and other races (*p* < 0.001) (Fig. 3A-3). Mexican Americans and other races showed higher midpoint of SD than NH whites with the difference reaching borderline significance for Mexican Americans (*p* = 0.055) and significance for other races (*p* = 0.029). There were no significant interactions between race and age observed.

As shown in Fig. 3B-1, social jetlag is present in early ages beginning almost at an average of one hour around age 6 and gradually increasing until around age 20. After around age 20, social jet lag steadily declines with age. There was a main effect of sex (*p* = 0.017) with males averaging 4 more minutes than females (Fig. 3B-2). There were no interactions between sex and age found. However, there also was a main effect of race (*p* = 0.001) which was driven by the significant difference (*p* < 0.001) between NH blacks and NH whites with NH blacks averaging 12 more minutes of social jet lag compared to NH whites (Fig. 3B-3). Mexican Americans and other races had higher values than NH whites and lower values than NH blacks in social jetlag, but the differences were not significant. There were no interactions observed between race and age.

## DISCUSSION

Within our study, we used actigraphy data to provide objective estimates of multiple sleep variability parameters: sleep duration SD, catch-up sleep, midpoint of sleep SD, and social jetlag. Our study is the first to show the distribution of these parameters in a nationally representative sample of the United States. We explored the differences in these parameters of sleep irregularity across the life span as well as the potential modifying effects of sex and race. We found that American youth and young adults as well as NH blacks had the largest variability across all sleep variability parameters compared to the other age groups or the other racial groups. The high rates of sleep variability in youth and young adults is especially concerning given its relationship with suicidal ideation and the observation that suicide death is the second leading cause of death among youth in the US^15^.

American young adults aged 18 to 25 had the highest sleep duration SD averaging 70 minutes which is consistent with a recent study on eight-pooled datasets which found that the 18–24 age group had the highest sleep duration SD averaging 77 minutes in the datasets using accelerometer recording^16^. Sleep duration SD showed a bimodal distribution with peaks around ages 20 and 70. This distribution held true when accounting for the impact of sex and race. Aging is associated with increased prevalence of multiple components which impact sleep such as multimorbidity, polypharmacy, psychosocial factors and many primary sleep disorders. All of which could contribute to increasing sleep duration SD after age 50^17^. The average age of retirement in 2013 was around 63 years old which also factors in as adults may taper off working hours leading into official retirement^18^.

The Multi-Ethnic Study of Atherosclerosis (MESA) in 1,922 participants aged 45–84 found that almost 40% participants had > 90 m of sleep duration SD and these participants had higher averages of BMI, blood pressure, and diabetes prevalence compared to those with < 90 m of sleep duration SD^4^. After adjusting for cardiovascular disease risk factors, this study also found that every 1h increase in sleep duration SD was associated with a 36% higher rate of cardiovascular risk^4^. Of the 3,917 participants over the age of 45 within the current study only 16% had > 90m of sleep duration SD. In addition to our study being conducted in a national representative sample, another potential reason for this difference could be that the previous study had different racial demographics. Of the participants of MESA 28% were African American compared to our study which had 11% NH Black participants. As shown in our study and prior research, NH Black Americans have greater amounts of sleep variability compared to other racial categories. Regardless, these findings illustrate the important impact of increasing sleep duration SD on the health of the geriatric population and emphases the need for ensuring public health education targeting on this topic. In addition, most research on the negative health impacts of sleep variability focus on older adults; however, our finding that 25% of participants aged 6–13 had 59 minutes of sleep duration SD highlights that variability in sleep pattern begins early in childhood. This warrants longitudinal studies to understand the impact of chronic sleep variability in childhood on different health parameters in later life.

In addition, our study examined the distribution of catch-up sleep across the lifespan. We found that 25% of participants aged 14–17 had greater than 2 hours of catch-up sleep which aligned with a previous study that reported 25.5% of adolescents in their study had > 120 minutes of catch-up sleep^19^. Prior literature has demonstrated that amounts of weekend catch-up sleep greater than 2 hours may be associated with mood and behavior disorders in adolescents^20^. Additional research is needed to understand the impact of different amounts of catch-up sleep on health through the lifespan.

Many factors like work and school schedules cause individuals to wake and sleep earlier during weekdays compared to weekends. This discrepancy is referred as social jetlag. We explored the distribution of social jetlag across all age groups. We found that social jetlag peaked around age 20 but all ages had varying amounts of social jetlag even young children. There is limited data on the quantity of social jetlag in various age groups; however, there have been studies which assessed total social jetlag in adolescents and in adults. A previous study used questionnaires to quantify social jetlag in non-shift workers aged 18–78 and found that almost 30% of participants had 1 to 2 hours of social jetlag^21^. In our study, the average social jetlag for participants aged 18–78 was 1 hour with 25% experiencing ≥ 1.5 hour social jetlag. In addition, a study of undergraduate students which used actigraphy estimated that students averaged about 40 minutes of social jetlag^22^. This is lower than our finding of an average of 1h 12m of social jetlag in the age group of 18–25 in our study. This discrepancy could be related to the small sample size (n = 84) of the earlier study and all the participants being university students.

Similar to the findings from the study on the eight-pooled datasets, we did not find a consistent difference between males and females in sleep variability parameters^16^. Sleep duration SD and catch-up sleep did not show significant differences between male and female; however, there was a significant main effect of sex on midpoint of sleep SD and social jetlag as males averaged greater amounts of each compared to females.

Current literature is growing on sleep through a social-environmental perspective. Multiple studies have indicated that race, as a social category, impacts sleep quality and quantity across the lifespan implicating race as a critical factor in current sleep disparities^23–25^. Many of these studies focused on comparisons between NH Black Americans and NH White Americans. However, as a nationally representative US sample, our study consisted of 65% NH White Americans, 11% NH Black Americans, 10% Mexican Americans and 14% of others. This allowed for reporting of sleep variability characteristics in Mexican Americans which is rarely cited in current research. We observed that Mexican Americans had significantly lower amounts of sleep duration SD, catch-up sleep, and midpoint sleep SD compared to NH Blacks. Mexican Americans also had significantly higher amounts of catch-up sleep compared to NH whites. In line with previous reports, NH Black Americans had worse sleep variability compared to all other racial groups. These findings align with a previous study which found that NH white youth had more sufficient sleep compared to NH black youth^24^. A multitude of factors may underlie the reasoning for these results. For instance, shift work tends to have variable shifts which could impact sleep variability, and people from minority racial group disproportionately hold shift work positions^25^. In addition, minority racial groups undergo an increased amount of daily stressors comparatively which is a potential underlying factor as stress is known to disrupt sleep patterns^25^. Moreover, a recent actigraphy study explored the relationship between racial disparities in sleep characteristics and hypertension prevalence and concluded that sleep maintenance mediated over 11% of the difference in hypertension prevalence between NH blacks and NH whites within their sample^26^. These findings highlight the significance for multifaceted approaches that include racial disparity when studying the impacts of sleep on health outcomes.

There are some limitations to this study. First, although accelerometer recordings provide objective measurements of sleep parameters, this method cannot detect a difference between lying still while awake or being asleep. Second, occupation status was not obtained from participants in the NHANES 2011–2014 cycle, therefore, exclusion criteria for certain occupations like shift workers could not be performed.

In conclusion, for the first time our study provides the distribution of multiple objectively measured sleep variability parameters over lifespan by sex and race in a US nationally representative sample. This study not only provided solid evidence on the high prevalence of irregular sleep patterns in the US population, but also indicated that irregular sleep begins in childhood and shows racial disparity. These findings provide understanding of sleep patterns for residents of the United States as well as emphasize the importance in future research on the social determinants of sleep from multiple aspects. Sleep variability is likely a modifiable risk factor for a variety of health conditions. This information can also further research and public health agendas on sleep hygiene.

## Figures and Tables

**Table 1:** General characteristics of the participants (n = 9,799)

Variables	Values
Age, years	41.9±21.5
Female, %	53.5
Race, %	
NH white	65.37
NH black	10.96
Mexican American	9.96
Other race	13.71
Sleep duration SD, hours	0.97±0.48
Catch-up sleep, hours	1.25±1.02
Midpoint of sleep SD, hours	0.70±0.39
Social jetlag, hours	1.03±0.84

For continuous traits, data are present as mean±SD.

NH Non-Hispanic.

**Table 2:** Distribution of sleep duration SD and catch-up sleep by age groups in minutes

Age Group	N	Sleep Duration SD						Catch-up Sleep					
		5%	10%	25%	50%	75%	90%	5%	10%	25%	50%	75%	90%
6–13	2,265	18	21	29	42	59	78	5	10	26	52	88	129
14–17	711	24	31	42	61	82	100	6	12	35	78	136	185
18–25	867	23	30	45	67	89	116	6	13	34	75	123	180
26–35	965	20	26	40	57	78	102	4	11	28	65	116	166
36–45	1,074	20	27	40	55	74	96	6	12	31	67	112	162
46–55	1,122	22	25	38	56	77	102	5	11	27	62	115	172
56–65	1,210	20	23	35	54	75	95	6	12	30	59	101	153
66–75	915	19	26	37	52	73	95	3	10	24	52	91	141
≥76	670	18	23	36	51	72	97	5	12	23	49	92	153

**Table 3:** Distribution of midpoint of sleep SD and social jetlag by age groups in minutes

Age Group	N	Midpoint of sleep SD						Social jetlag					
		5%	10%	25%	50%	75%	90%	5%	10%	25%	50%	75%	90%
6–13	2,265	12	14	22	32	47	64	5	11	27	54	93	133
14–17	711	17	21	32	44	61	82	6	14	36	82	120	160
18–25	867	16	21	30	46	66	87	6	11	24	58	110	164
26–35	965	17	20	28	42	60	80	6	10	26	54	102	152
36–45	1,074	14	18	26	38	54	73	5	9	24	53	93	138
46–55	1,122	14	18	27	38	53	70	6	10	23	52	94	130
56–65	1,210	13	16	23	34	50	67	3	7	20	42	75	114
66–75	915	11	14	20	32	46	67	2	5	12	32	60	98
≥76	670	12	15	21	31	45	63	3	6	15	32	61	91

## Data Availability

All data are publicly and freely available from the US Centers for Disease Control and Prevention’s National Center for Health Statistics and can be accessed at https://www.cdc.gov/nchs/nhanes/index.htm
